# Motion‐ and Field‐Robust Mesoscopic Whole‐Brain $T_{2}^{*}$‐Weighted Imaging at 7 and 11.7 T Using Servo Navigation

**DOI:** 10.1002/mrm.70251

**Published:** 2026-01-12

**Authors:** Matthias Serger, Rüdiger Stirnberg, Philipp Ehses, Malte Riedel, Thomas Ulrich, Caroline Le Ster, Franck Mauconduit, Vincent Gras, Alexis Amadon, Alexandre Vignaud, Son Chu, Shajan Gunamony, Maxim Zaitsev, Nicolas Boulant, Klaas P. Pruessmann, Tony Stoecker

**Affiliations:** ^1^ MR Physics, German Center for Neurodegenerative Diseases (DZNE) Bonn Germany; ^2^ Department of Physics & Astronomy University of Bonn Bonn Germany; ^3^ Institute for Biomedical Engineering, ETH Zurich and University of Zurich Zurich Switzerland; ^4^ CEA, CNRS, BAOBAB, NeuroSpin, University of Paris‐Saclay Gif sur Yvette France; ^5^ Imaging Centre of Excellence University of Glasgow Glasgow UK; ^6^ Division of Medical Physics, Department of Diagnostic and Interventional Radiology, University Medical Center Freiburg, Faculty of Medicine University of Freiburg Freiburg Germany

**Keywords:** 11.7 T, 3D‐EPI, brain vasculature, mesoscopic imaging, prospective motion correction, servo navigation

## Abstract

**Purpose:**

To mitigate artifacts related to motion and field changes in high‐resolution $T_{2}^{*}$‐weighted human brain imaging using servo navigation at ultra‐high fields up to 11.7 T.

**Methods:**

MR‐based servo navigators were integrated into a segmented 3D‐EPI sequence to allow for prospective correction of involuntary head motion and first‐order shim changes. Seven subjects were scanned with whole‐brain protocols at 0.3 mm isotropic resolution with and without correction at 7 and 11.7 T. Validation was performed on detailed brain vasculature in scans with involuntary motion.

**Results:**

Blurring of small veins was reduced by servo navigation for all subjects and across field strengths. In case of involuntary large motion, the method preserved image quality, while uncorrected motion led to severe artifacts. In case of microscopic motion, reduced blurring and shading in the frontal lobe demonstrate the additional benefit of prospective field drift correction.

**Conclusion:**

Servo‐navigated segmented 3D‐EPI improves 0.3 mm isotropic whole‐brain $T_{2}^{*}$‐weighted imaging under realistic motion and field changes within 5.5 to 11 min scan time at 11.7 and 7 T.

## 1 Introduction

The rapid development of ultra‐high field (UHF) MRI systems in the past decades pushed the SNR to unprecedented levels, enabling the acquisition of ultra‐high‐resolution whole‐brain images with voxel sizes in the mesoscopic regime (≤ 0.5 mm isotropic) in less than an hour. With such small voxels, even involuntary small motion can lead to image artifacts (e.g., blurring) that defeat the nominal high resolution. Additionally, long scan times increase patient discomfort, thus enhancing the probability of motion. The demands on motion correction methods increase, as the precision needs to be several times higher than the image resolution [1, 2]. Some external tracking systems based on markers (e.g., Moiré Phase Trackers [3, 4], NMR markers [5, 6, 7], short wave trackers [8]) demonstrated to be capable of providing highly precise and temporally resolved motion estimates but require additional hardware and preparation steps, potentially causing additional patient discomfort. Alternatively, motion can be tracked by interleaved MR measurements (navigators) using the scanner hardware. Image‐space navigators (volumetric [9] or fat navigators [10, 11]) fulfill the requirements in terms of spatial precision, but the temporal resolution is low or trades off against scan time. On the other end of the spectrum, ultra‐short FID navigators (FIDNavs [12, 13]) require extensive calibration and lack precision [14] suitable for mesoscopic imaging.

As an intermediate approach, k‐space navigators leverage the knowledge that rigid body head rotation and translation result in rotations and linear phase ramps in k‐space, respectively. Many navigator designs were proposed and evolved from simple linear [15] and orbital navigators [16, 17] to more sophisticated designs like cloverleafs [18]. Apart from the improvements in accuracy, precision and acquisition time, the estimation of head rotations remained a critical step due to projection errors made when rotations occur about axes not probed by the navigator trajectory. For estimating off‐axis rotations by cloverleaf navigators, for instance, a 12 s motion‐free pre‐scan was proposed to map the angular k‐space neighborhood [18]. Full‐shell coverage can be achieved by spherical navigators [19] that either require computationally expensive fitting algorithms or a pre‐scan with rotated shells to create a lookup table [20]. Improvements were achieved by Lissajous sampling [21] that reduces the acquisition time and thus computational cost; however, the small k‐space radii used (63 rad/m) provide relatively low angular sensitivity.

Recently, a new prospective motion correction (PMC) technique termed servo navigation [22, 23] was established that overcomes many of the drawbacks of previous methods, providing high precision and angular sensitivity, low computational cost, short acquisition and calibration time. It is based on modeling small motion‐ and field‐induced k‐space changes as linear perturbations, expressed by the linear terms of a Taylor expansion of the complex‐valued signal equation with respect to rigid‐body motion and field changes. To detect these changes, a short single‐shot 3D orbital k‐space trajectory (e.g., 2.3 ms) is acquired and compared to a single reference navigator scan. Motion and field estimates provided by the linear model are then used to update the trajectory (and image geometry), RF frequency and shim setting to stay close to the reference, ensuring k‐space consistency and thus, motion correction. This negative feedback loop (servo control) has proved to be stable and increases the linear range of the model, maintaining high accuracy for motions up to $∼2^{\circ }$ and 2 mm in spin‐warp and single‐shot 3D‐EPI sequences [22, 24]. For cooperative subjects, this range of motion is usually not exceeded.

In addition to inconsistencies in spatial encoding, head motion causes changes of its susceptibility‐induced field, predominantly with rotations orthogonal to the main field axis (e.g., $R_{x}$ and $R_{y}$ if $B_{0}$ ‖ z). Especially in regions with high susceptibility differences (e.g., air‐tissue interfaces, ear canals), these field changes are spatially non‐linear [25] and may lead to ringing and blurring artifacts. They can be corrected retrospectively, for example by data‐driven approaches [26] or based on dual‐echo volumetric navigators [27] that provide spatially‐resolved $B_{0}$ sensitivity at the expense of prolonged measurement times. These navigators recently demonstrated reduced motion and field artifacts in mesoscopic 3D‐EPI (0.4 mm iso.) at 10.5 T [28, 29]. However, prospective correction of non‐linear field variations requires dynamic switching of higher‐order shim coils, which is not supported by some current MR systems and remains technically challenging due to long‐lived eddy currents and the lack of active shielding in typical shim coil designs [30, 31]. On the other hand, dynamic shimming of linear fields using the scanner gradient system is possible at high temporal resolution. This has been demonstrated to permit expansion of servo navigation to joint correction of motion and first‐order field perturbation [23], which offers high sensitivity and precision of gradient offset estimates. It allows to correct, to first order in space, for physiological field fluctuations, susceptibility‐induced field changes, and field drift.

This work demonstrates an application of servo navigation for mesoscopic whole‐brain $T_{2}^{*}$‐weighted imaging at 7 and 11.7 T [32]. To this end, the method is integrated into a segmented 3D‐EPI sequence [33] that renders relatively short scan times at maximum SNR efficiency feasible. Compared to line‐by‐line gradient echo imaging, the short scan time already offers a moderate increase of robustness against motion artifacts. However, fast gradient switching in 3D‐EPI results in gradient heating that causes additional field drifts. In combination with typically long echo times for enhanced $T_{2}^{*}$ contrast, the sensitivity to $B_{0}$ artifacts is increased. Therefore, servo navigation is not only an interesting candidate for correcting intra‐volume subject motion and motion‐induced field changes in high‐resolution anatomical 3D‐EPI imaging, but also for compensating dynamic field changes generated by the host sequence itself.

To validate this method, multiple subjects were scanned with and without servo navigation at 7 T using whole‐brain protocols at 0.3 mm isotropic resolution acquired in 11 min. The datasets were analyzed qualitatively with respect to motion and field artifacts in the brain vasculature, especially in small blood vessels that are referred to as meso‐veins in recent literature [34]. Additional experiments with the same spatial resolution were conducted in reduced scan time (5.5 min) at 11.7 T.

## 2 Methods

### 2.1 Servo Navigation in Segmented 3D‐EPI

**FIGURE 1:**
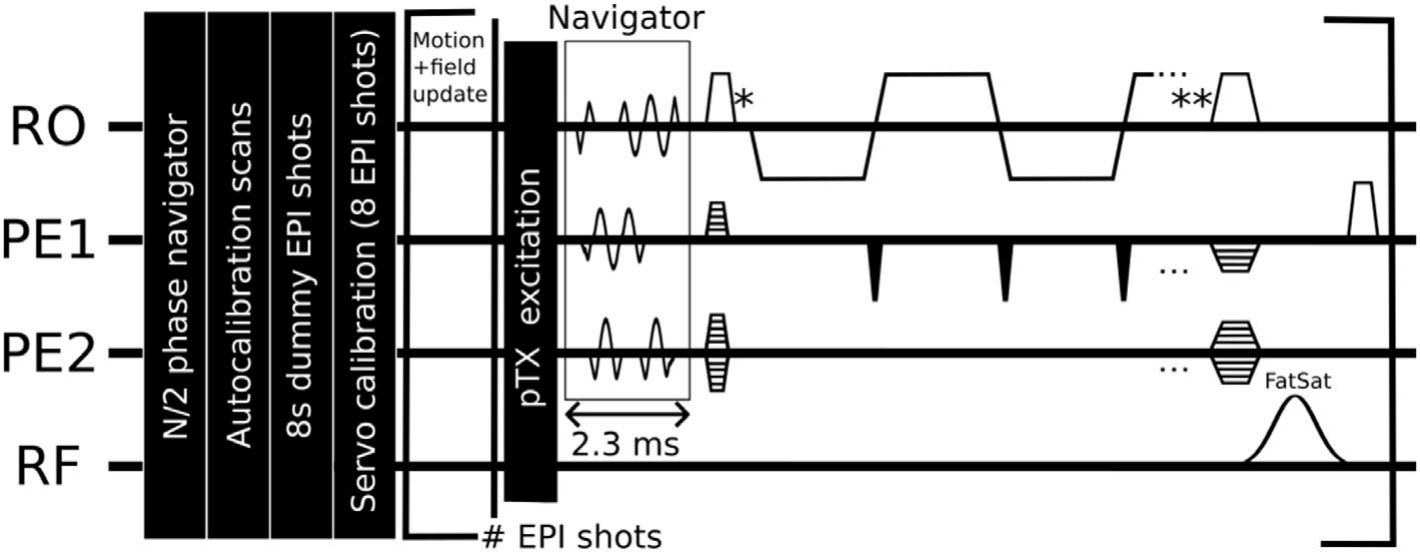
Modified 3D‐EPI sequence featuring a 3D orbital k‐space navigator readout immediately following RF excitation (pTX slab selection). Three external phase navigator lines are acquired once per measurement to estimate the odd/even echo displacement (N/2 ghost correction). Fat saturation was applied only at 7 T. Pre‐(*)/post‐(**)‐EPI delay times indicate echo time shifting.

A single‐shot 3D orbital k‐space trajectory [22] (400 rad/m, 2.3 ms duration) was incorporated into a pTx‐enabled skipped‐CAIPI [35] 3D‐EPI sequence [33], inserted between the RF excitation pulse and EPI readout (Figure 1). Following 8 s of dummy EPI shots to establish a steady‐state, eight calibration shots were acquired: two reference, three shots with rotated trajectories (0.5° around *x*, *y*, *z*) and three shots with 5 μ T/m gradient offsets ($G_{x}$, $G_{y}$, $G_{z}$). The finite differences method [22, 23] was used to derive signal changes with respect to the first reference navigator upon rotation and linear field changes, while analytical derivations for translations and $B_{0}$ 0th order terms (phase and frequency offset $f_{0}$) were calculated to fill the columns of the model matrix. After computing its pseudo‐inverse, signal changes with respect to the second reference navigator were multiplied to the inverted matrix to obtain motion and field changes. The model was established with two separate reference navigators to avoid parameter estimation offsets [23]. For servo control, the RF frequency, shim setting, and navigator geometry were updated at run time, before each RF excitation pulse. This requires fast processing that is described in the following section.

While navigator data were also collected in uncorrected scans, the servo control had to be disabled—that is, the trajectory frame of reference, RF frequency and shim settings stayed constant—which limits the linear model range and biases the motion parameter estimation in case of larger motions. Nevertheless, detection of large motion and accurate tracking of small motion were still feasible for the sake of comparing corrected data versus non‐corrected data across two separate scans.

### 2.2 Run‐Time Motion and Field Correction

**FIGURE 2:**
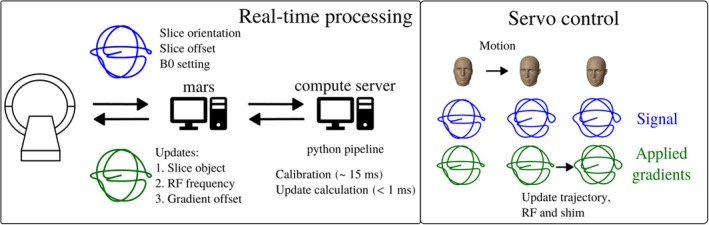
Workflow for servo navigation on a Siemens system. Navigator data are streamed from the scanner to a Python pipeline on a compute server, which calibrates the linear model and computes motion and field updates. It also tracks the current slice position and $B_{0}$ setting (received with the navigator) to calculate updates relative to the reference frame.

The navigation method, originally developed on a Philips system, was implemented on a Siemens (Healthineers, Erlangen, Germany) MRI platform by establishing a real‐time processing workflow that is depicted in Figure 2. In this setup, navigator data were streamed from the acquisition and reconstruction server (mars) to a dedicated compute server [14]. Once all calibration shots were received, the model was calibrated (15 ms), and updates could be computed rapidly (< 1 ms), enabling prospective corrections with every EPI shot (every ∼50 ms). The total system latency was estimated at 5–6 ms, including a minimum delay of 2.5 ms needed by the vendor's measurement system to prepare the sequence for real‐time execution. To enable servo control, the current slice position and $B_{0}$ settings were transmitted along with the navigator data to compute updates relative to the reference navigator, following the approach described by Ulrich et al. [22] and Riedel et al. [23] To ensure compatibility with the motion correction library (libXPACE [3]), motion parameters were transformed from gradient coordinates PRS (Phase‐Read‐Slice) to scanner device coordinates XYZ (corresponding to Left‐Anterior‐Inferior with head‐first/supine patient positioning). Next, the frequency and gradient offset updates, received alongside motion updates from libXPACE, were used to adjust the RF (de)modulation frequency and the first‐order shim terms. Due to hardware constraints, linear shim updates could only be applied in discrete increments of ∼0.14 μ T/m.

### 2.3 Parameter Bias Correction

Rapid gradient switching in 3D‐EPI imaging causes mechanical oscillations of the gradient system and eddy currents that change with the encoding gradients. This leads to varying fields during the navigator readout and degrades its precision for motion and field estimation, especially if short‐term field fluctuations are present that differ between navigator and EPI readout. Two major sources of systematic parameter biases related to the 3D‐EPI's gradient encoding were identified: 1. echo time shifting (ETS [36]), which varies from shot to shot along with small changes in the first phase encoding moment (PE1), and 2. the slowly varying second phase encoding rewinder gradient (PE2_rew_). While the latter is fundamental for spatial encoding in slice direction in 3D‐EPI, echo time shifting is typically employed with in‐plane segmentation.

Since the time between the end of the shifted EPI train and the subsequent shot navigator varies with ETS, eddy‐currents induced by the shifted EPI gradients vary during the navigator and lead to biased predictions. The prediction bias pattern repeats every S shots, reflecting the repetition of specific echo time shifts with each step in the second phase encoding direction (partition). To determine these biases, the predictions during the first four partitions are averaged per in‐plane shot to suppress motion and field fluctuations. Then, these shot‐specific biases are subtracted from subsequent predictions at run‐time. The bias estimates are recalculated from the uncorrected predictions every new partition in a sliding window fashion (including the last four partition shots) to account for bias changes by the PE2_rew_ gradient.

This phase‐encoding gradient varies slowly throughout the measurement, and the strength of its associated eddy‐currents during the navigator readout depends on gradient shape, system characteristics, and timing. In this work, the gradient impulse response function [37] (GIRF) was used to characterize field oscillations of the PE2_rew_ gradient in terms of amplitude and phase, and to reduce their impact on navigator data. Oscillations during the navigator readout were reduced by carefully adapting the PE2_rew_ gradient waveform. In some experiments, the main lobe of its frequency response was narrowed so that its first zero crossing aligned with the lowest acoustic resonance frequency of the gradient system. Furthermore, side lobes of the frequency response were minimized by adjusting the ratio between the ramps and flat‐top of the trapezoidal gradient (roll‐off factor set to 2/3). Gradient shaping was especially important to minimize acoustic resonances of the z‐Gradient at 11.7 T [38].

The precision of the prediction and the effectiveness of the correction were validated in phantom and in vivo measurements by calculating the standard deviation after applying a high‐pass filter to remove drifts (cf. tab. 2 in Riedel et al. [23]). In order to capture the repeating ETS pattern, the threshold frequency of the filter was set to 1/(TR × *S*). For estimating the in vivo precision, this relatively low threshold frequency may cause bias by abrupt or fast oscillatory (cardiac or respiratory) motion which is not sufficiently suppressed.

### 2.4 Experiments

Two segmented whole‐brain 3D‐EPI protocols with the same isotropic resolution of 0.3 mm were set up at 7 T (I) and 11.7 T (II). The protocol parameters are summarized in Table 1. For the 11.7 T protocol, the echo time and EPI factor were slightly reduced to obtain a similar contrast as for Protocol I at 7 T. As 11.7 T provided higher SNR [39], stronger parallel imaging undersampling could be employed, resulting in a scan time of only 5.5 min. At both field strengths, a four‐spokes slab‐selective pTX pulse was used for excitation [40]. At 7 T, a universal pulse [41] was calculated based on a *B*_0_/*B*_1_ database of 21 subjects. At 11.7 T, a subject‐specific pulse was computed.

**TABLE 1:** Segmented 3D‐EPI protocols for mesoscopic imaging at 7 and 11.7 T.

Protocol	I	II
Field strength	7 T	11.7 T (7 T)^a^
Iso. voxel [mm]	0.3	0.3
Matrix (LR × AP)	484 × 720	484 × 720
Slices (HF)	460	460
Slice oversampling [%]	10.0	5.2
TE [ms]	23	18.7 (19.0)
TR [ms]	54.1	43
Nominal FA [°]	12	12
Phase PF	0.93	0.92
Parallel Imaging	1 × 2	2 × 2
Slice CAIPI shift	1	1
Fat saturation	Yes	No
Seg./EPI f.	48/14	30/11
Echo spacing [ms]	2.4	2.32 (2.44)
RO bandw. [Hz/mm]	1640	1717 (1640)
PE bandw. [Hz/mm]	93	120 (114)
TA [min: s]	11:20	5:28

^a^ 11.7 T protocol replicated at 7 T (minimal adaptations required).

#### 2.4.1 Phantom Experiments

Phantom scans using protocols I and II were conducted to validate the parameter bias correction and the motion and field estimation precision at 7 and 11.7 T, respectively.

The accuracy of servo navigation at 11.7 T was first evaluated in phantom scans using a 3D‐EPI time series protocol (1.2 mm iso., TR = 2.12 s, TR = 58 ms). In order to validate the motion correction, an anthropomorphic head phantom was moved abruptly by a stick from outside the bore, and the residual motion was assessed by retrospective image registration [42]. The prospective field correction, on the other hand, was evaluated by moving a bottle phantom towards the head phantom to induce a low‐order field change from outside the FoV.

#### 2.4.2 In Vivo Experiments

Four healthy subjects were scanned using a MAGNETOM 7 T Plus scanner (Siemens Healthineers, Erlangen, Germany) equipped with a 32 channel Rx (8Tx) head coil (Nova Medical Inc., Wilmington, MA, USA). All subjects were instructed to remain still during the scans, and two measurements, with and without motion and field correction, were acquired per subject with alternating scan orders. Protocol I was applied in three subject scans (Table 1), while Subject 4 was scanned with the replicated 11.7 T protocol.

At the 11.7 T scanner [32] (ISEULT, Neurospin, CEA, Paris, France), three additional subjects (5–7) were scanned with a custom 31 channel Rx (8Tx) head coil [43, 44] using Protocol II (Table 1).

All participants provided written informed consent in accordance with local ethics regulations at each scanning site.

All magnitude and phase images were reconstructed using the vendor image reconstruction applying 3D GRAPPA kernels (“IcePAT”) and a virtual reference coil‐based adaptive coil combine method [45]. Since both magnitude and phase images were of primary interest and partial Fourier factors were modest, we relied on tapered zero‐filling as the only available option provided by “IcePAT.”

### 2.5 Preprocessing

Magnitude images were bias field corrected using ANTsPy [46, 47]. A complex‐valued adaptation [33] of nonlocal means denoising [48] was applied on magnitude and phase data used for SWI and QSM. Apart from that, magnitude images were not denoised. Magnitude images acquired with and without prospective correction were registered (MCFLIRT [42]) and transformed to halfway space via spline interpolation [49]. SWI images were generated using Clear‐SWI [50] (https://github.com/korbinian90/CLEARSWI.jl) and QSM maps using a Julia QSM toolbox (https://github.com/kamesy/QSM.jl), then coregistered using the magnitude‐based halfway space transforms. Brain vasculature was visualized using minimum‐intensity projections (minIPs) of 10 mm slabs of magnitude and SWI images. The paramagnetic effect of small blood vessels (due to deoxygenated blood) was further visualized by QSM venograms in maximum‐intensity projections (MIPs) of a 25 mm slab. To enhance vessel visibility in the QSM venograms, a high‐pass filter was applied for the background phase removal [51] prior to dipole inversion, by selecting a smaller starting radius (3 mm) for the spherical mean value kernel [52].

## 3 Results

### 3.1 Tracking Precision

**FIGURE 3:**
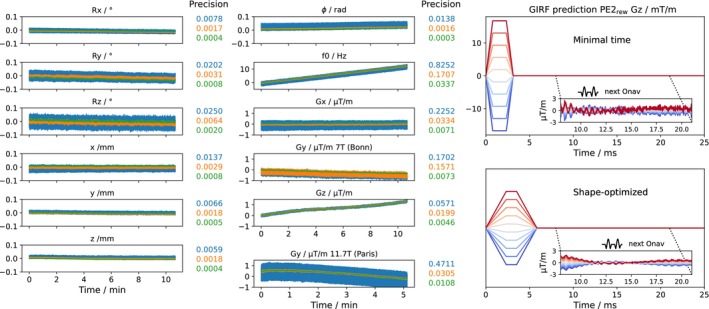
Parameter bias correction for servo navigation in 3D‐EPI. Left: Correction of fast oscillating fields caused by echo time shifting. Colored numbers indicate high‐pass‐filtered standard deviations. Blue and orange lines represent parameters before and after the bias correction, respectively; green shows results after applying both parameter correction and the moving average filter as used in vivo. Right: Mitigation of PE2 rewinder field fluctuations through gradient shaping. The estimates shown in the left panel correspond to the shape‐optimized protocol.

In Figure 3, the effectiveness of the parameter bias correction is demonstrated for Protocol I at 7 T. Motion parameter precision is improved significantly, from levels of up to 25 mdeg and 14 μm down to below 7 mdeg and 3 μm. Substantial improvements were also observed for the field parameter estimates, except for $G_{y}$. Interestingly, the insufficient *y*‐gradient offset correction observed in the 7 T phantom data was not present in the 11.7 T data. To improve robustness against outliers and in vivo imperfections, a moving average filter was applied in practice and evaluated in the phantom experiment. The filter window was set to 10 (=0.54 s) for motion parameters and 14 (=0.76 s) for field parameters, balancing high precision with relatively low latency. This effective low‐pass filtering improved parameter estimate precision by at least a factor of three in the absence of actual motion (Figure 3). A corresponding analysis of in vivo precision across all subjects is presented in Figure S1.

In addition to fast echo time shifting‐related oscillations, a systematic drift in the navigator's *z*‐gradient offset of ∼1 μ T/m prediction was observed. This drift is characteristic of variations in the PE2_rew_ gradient during the measurements. However, the GIRF prediction (Figure 3, right) indicates that the navigator is already placed in a region of small field fluctuations induced by the rewinder and thus, the bias cannot be further reduced by shifting the navigator readout, for example.

#### 3.1.1 Accuracy at 11.7 T.

Registered motion parameters of the moving phantom scans at 11.7 T are depicted in Figure S2. Abrupt motion ($R_{x}\approx -1.9^{\circ }$ and $T_{z}\approx -2.3$ mm) was corrected with little residual registered motion ($∼0.03^{\circ }$ and 0.04 mm). The field change introduced by the bottle (second phantom experiment) caused an apparent shift in the low phase encoding bandwidth direction (*y*) of the time‐series protocol, which was effectively mitigated by prospective field correction.

### 3.2 Mesoscopic Imaging at 7 T

**FIGURE 4:**
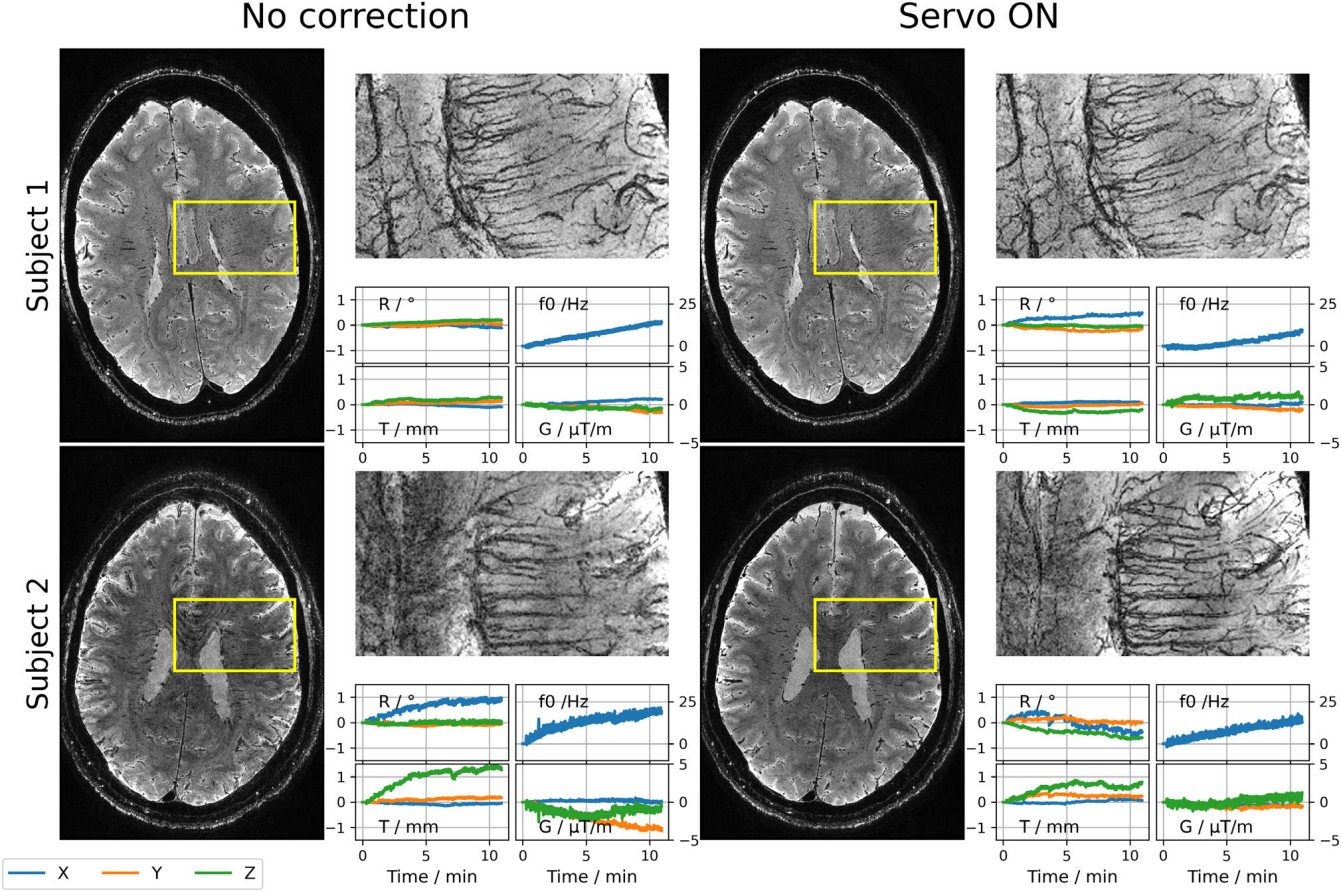
Representative 7 T data sets (Subjects 1–2). Subject 2 exhibited larger motions in both scans, with a clear reduction of blurring artifacts in the corrected image. Subject 1 moved more in the corrected scan, but image quality is preserved.

**FIGURE 5:**
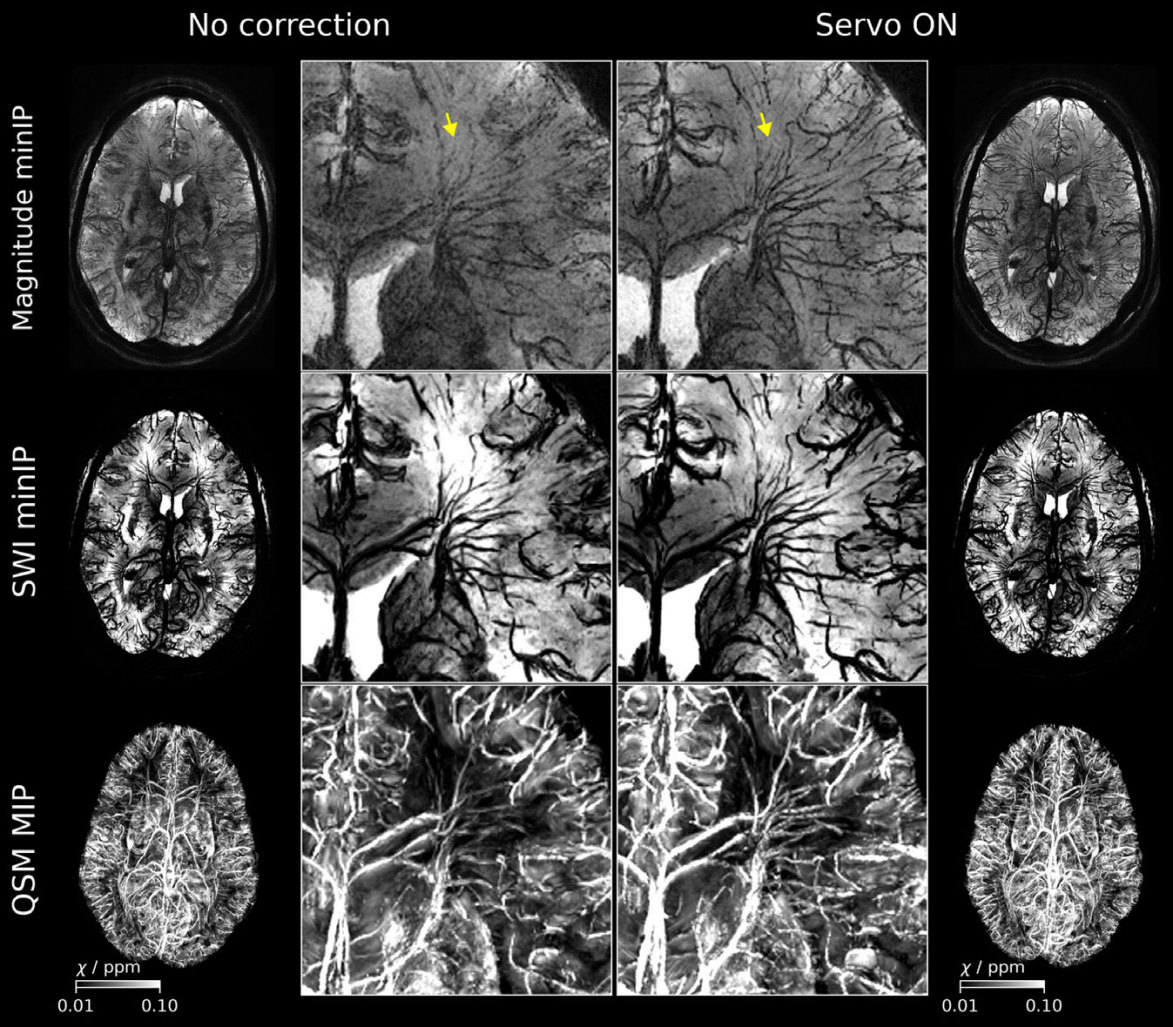
SWI minIPs and QSM venograms of Subject 2 without and with servo navigation. Uncorrected SWI minIPs show reduced blurring artifacts of small vessels compared to magnitude minIPs but introduce blooming around large veins. QSM venograms (MIPs) display less vessel blurring than magnitude images. Clear improvements in white matter vein visibility are observed with correction (yellow arrows).

Three orthorgonal slices of all subjects scanned at 7 T are depicted in Figure S3. Figure 4 shows two representative 7 T data sets, comparing scans without (left) and with prospective correction (right). The most severe artifacts are observed in the magnitude minIP of the uncorrected scan of Subject 2, while image quality is substantially improved in the scan using servo navigation. Furthermore, increased ringing artifacts can be observed in the frontal lobe of the uncorrected magnitude image. However, it is important to note that especially nodding motion ($R_{x}$) was more pronounced in the uncorrected scan. In contrast to that, Subject 1 exhibited larger nodding motion in the corrected scan, but image quality is preserved. Subjects 3 and 4 scanned at 7 T exhibited little and comparable motion during both scans (Figure S3). QSM venograms, SWI and magnitude minIPs of Subject 2 are depicted in Figure 5. Reduction of blurring artifacts can be observed in the corrected magnitude image as well as in the SWI miniP and QSM venogram. Notably, the SWI minIP and QSM venogram of the uncorrected scan retain slightly more detail than the uncorrected magnitude minIP. While pronounced blooming artifacts of vessels can be observed in SWI minIPs compared to magnitude minIPs, QSM venograms reveal fine vessels. Corresponding SWI minIPs and QSM venograms of Subject 3, who exhibited smaller motion during both scans, are shown in Figure S4. Improvements in the visibility of white matter medullary veins can be observed but are less pronounced than for Subject 2.

### 3.3 Mesoscopic Imaging at 11.7 T

**FIGURE 6:**
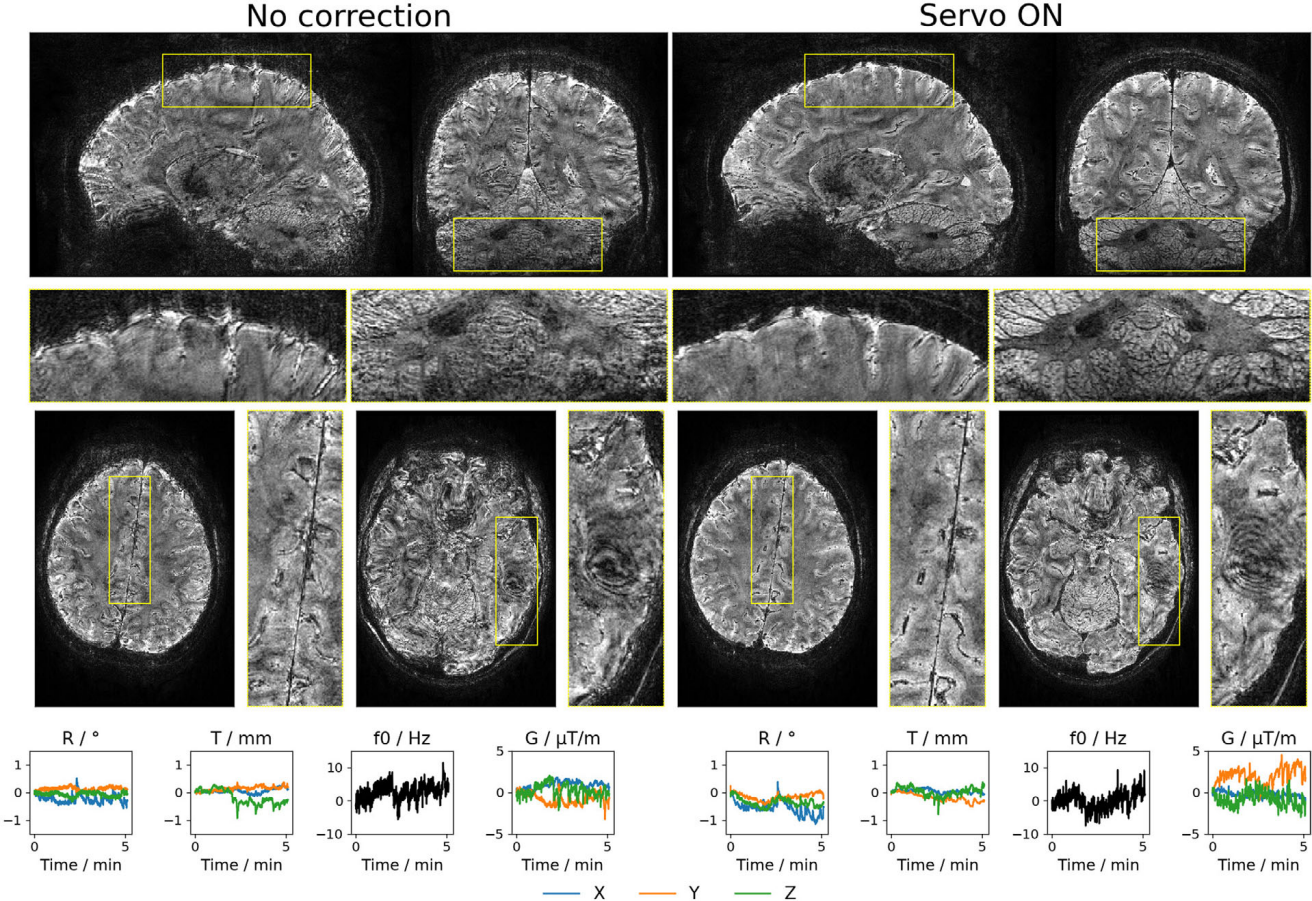
Sagittal, coronal, and two axial slices of the second subject scanned at 11.7 T (Subject 6), who performed similar rapid large motions in both scans near the k‐space center. Zoomed views of the cortex and cerebellum reveal a clear reduction in blurring artifacts in the corrected image. Interference artifacts in the temporal lobes above the ear canals remain in both images.

Magnitude images of all subjects scanned at 11.7 T are presented in Figure S5. Figure 6 shows magnitude images along with motion and field parameters of Subject 6, the second participant scanned at 11.7 T. Although the subject performed moderate motion in terms of magnitude ($<1^{\circ }$ and < 1 mm), several rapid motion events were detected in both scans. Notably, during the k‐space center acquisition, a nodding motion ($R_{x}∼0.5^{\circ }$) is visible in both motion traces, allowing for a balanced comparison. Zoomed views of the cortex in the sagittal slice and the cerebellum reveal substantially reduced blurring artifacts in the corrected images. Additional zoom‐ins of an upper axial slice highlight reduced blurring in the longitudinal fissure and small peripheral veins. Despite these improvements, $B_{0}$‐related interference artifacts remain visible in the right temporal lobe above the ear canals in both images.

**FIGURE 7:**
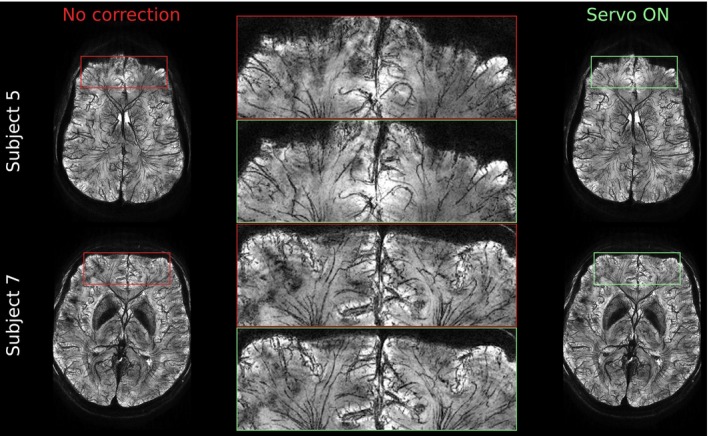
Axial minIPs of two subjects scanned at 11.7 T. Both exhibited only small motions, resulting in subtle artifacts. Improvements by servo navigation, mainly due to prospective first‐order shimming, are most noticeable in the frontal lobe, where shading and blurring of meso‐veins are reduced.

The remaining two subjects (5 and 7) scanned at 11.7 T only moved little during both scans (Figure S5) and differences in the magnitude minIPs, both showing high detail, are hardly visible (Figure 7). However, in the frontal lobe, which is particularly sensitive to susceptibility‐related $B_{0}$ changes, meso‐veins appear less blurred and shading is reduced in the corrected images.

**FIGURE 8:**
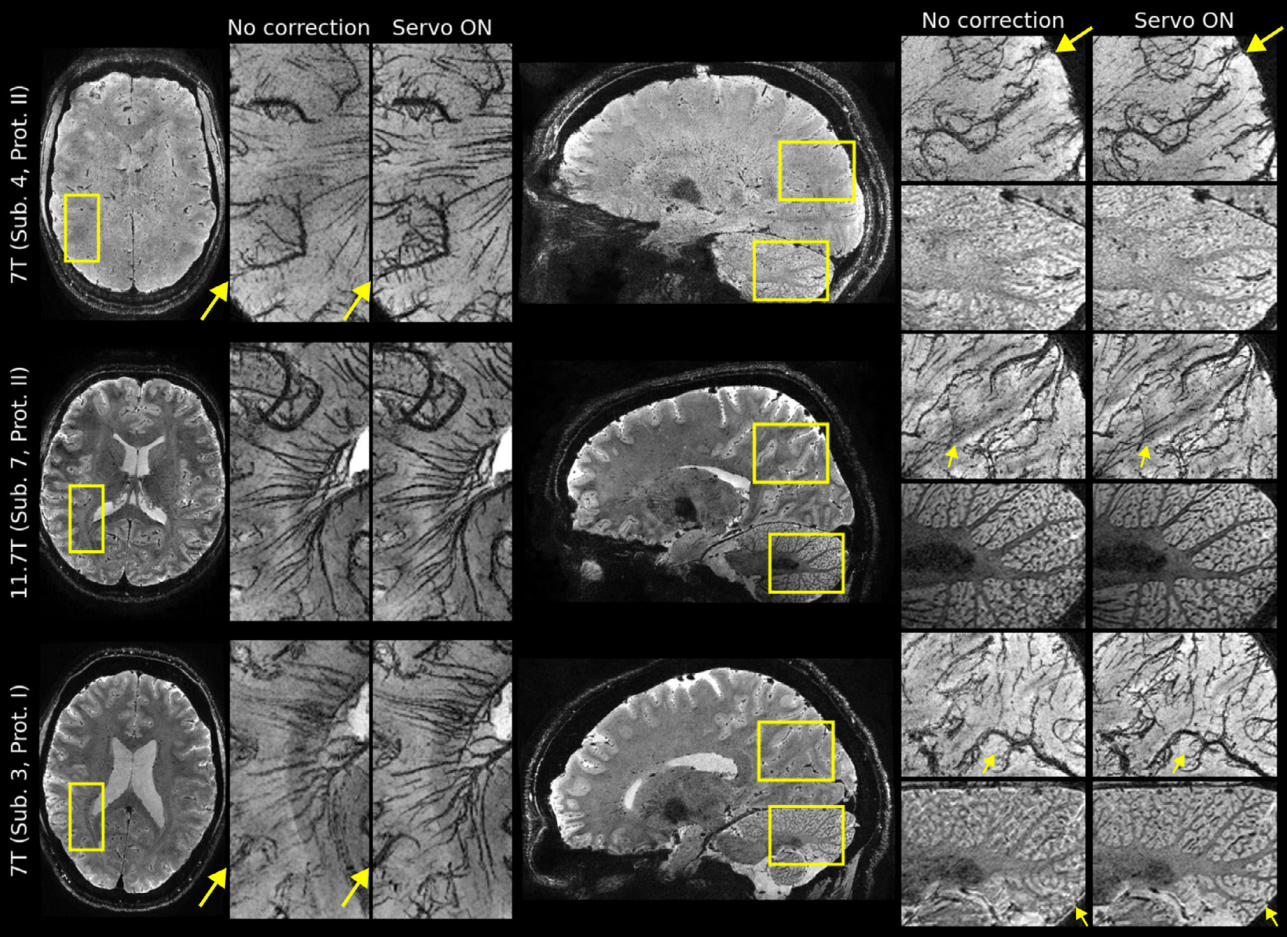
Comparison of equivalent 0.3 mm isotropic scans at 7 T and 11.7 T. Zoomed views show 10 mm magnitude minIPs, except for the sagittal view of the cerebellum, which displays the raw magnitude images like the whole‐head views. Reduced motion blurring artifacts can be observed across all scans (yellow arrows).

Figure 8 compares several datasets acquired at 7 and 11.7 T (cf. Experiments I and II in Table 1). Although the protocol optimized for 11.7 T offers reduced contrast and SNR at 7 T (Subject 4), the minIPs still exhibit strong vein‐tissue contrast. Across all scans, high detail and reduced blurring artifacts can be observed in meso‐veins in the corrected images.

## 4 Discussion

### 4.1 Tracking Precision

Predicting motion and field parameters from orbital servo navigators embedded in host sequences that rapidly switch strong gradients, such as segmented 3D‐EPI, remains challenging. Although the proposed parameter bias correction significantly improved prediction precision, the single‐digit mdeg/μm and 30 nT/m gradient offset precision previously reported for 3D‐GRE sequences [22, 23] could only be achieved in in vivo scans with additional filtering in this study. Residual fluctuation was still correlated with phase encoding, suggesting a key role of stronger phase encoders for high resolution along with potential differences in gradient hardware and eddy current correction on the spectrometers. The proposed correction method is limited to mitigating only systematic offsets that repeat along with the echo time shifting pattern. At 7 T, unlike at 11.7 T, the correction was not effective for the y‐shim correction. This indicates that the observed *G*_y_ oscillations were not dominated by a systematic ETS pattern, unlike other parameters. This deviation seems to be specific to the 7 T system used. Furthermore, the precision of the bias correction was lower in vivo than in phantom scans. This may be attributed either to residual subject motion not fully removed by the low‐threshold high‐pass filtering, or to motion and field changes occurring during the parameter bias estimation window that could not be averaged out completely. As a result, applying an additional parameter filter became necessary to ensure sufficient precision at run‐time. The simple moving average filter used in this work introduces temporal smoothing and a delay in updates, which limits the ability to correct for rapid motion or physiological fluctuations, such as respiratory oscillations.

Furthermore, the run‐time bias calculation may be less accurate if large motions are not averaged out. Figure S6 illustrates simulated motion data for rapid pose changes (0.5°, 1°, or 2°) and different motion velocities. In the extreme motion case (0.77°/s), the bias corrected time series shows an oscillation of approx. 0.25° after the motion event which is not present in the raw prediction and subsides afterwards, indicating that the bias correction introduced this error. This was however not observed for the 0.5° motion of Subject 6 (Figure S1). In the future, the influence of such motion events could be mitigated by excluding affected shots or partitions from the bias calculation using a metric like the RMS deviation [42].

Although field fluctuations in the navigator signal are sequence‐driven to a large extent, the proposed parameter bias correction depends on the calibrated reference head position and the shim setting. The proposed characterization by retrospective filtering intrinsically lacks information about the sequence history and its eddy currents for a more generic correction, making a scan‐specific calibration necessary for this method. Alternatively, making use of eddy current simulations to actively adapt the acquisition could make navigator predictions more robust against sequence‐driven field variations. However, this is beyond the scope of this work.

The scans of the abruptly moving phantom in a 3D‐EPI time‐series scan at 11.7 T demonstrated an effective correction of a ∼2.3 mm and ∼1.9° motion with only ∼0.04 mm and ∼0.03° residual (uncorrected) motion. This suggests that the tracking accuracy range originally reported for 7 T [22] can be maintained at this field strength using a comparable receive head coil array.

### 4.2 Artifact Reduction by Servo Navigation

Across multiple subjects and field strengths, servo navigation consistently reduced motion‐ and field‐related artifacts. In cases of involuntary large motion (e.g., Subject 2 and 6), the method largely preserved high image quality while uncorrected images with similar motion traces revealed severe artifacts that disqualify such images from further analysis. Nevertheless, pose‐dependent susceptibility‐induced fields remain a problem (cf. Figure 6) that becomes more pronounced as field strength increases. Subtle improvements due to prospective first‐order shimming were still possible at 11.7 T (cf. Figure 7). In the future, this problem could be tackled at the reconstruction stage [26, 53] or by prospective higher‐order shimming [30, 54, 55, 56, 57]. As discussed by Riedel et al. [23], the linear model could be extended to include non‐linear field terms using the finite differences calibration method at the cost of prolonging the calibration by ∼200–300 ms (e.g., by five additional shots for second order spherical harmonics). In order to maintain high precision for higher‐order B0 terms, longer navigators may be beneficial to enhance the sensitivity by increased phase accumulation.

The impact of motion‐induced higher‐order field changes on image quality was not systematically investigated in this work. However, residual blurring and shading artifacts visible in the corrected image of Subject 6 at 11.7 T (Figure S5) may be an indicator for non‐negligible remaining B0 variations. Especially, the differences in shading between both scans may be attributed to susceptibility‐induced higher‐order field changes under slightly different poses. In contrast to that, similar shading artifacts in the minIPs of Subject 7 (left hemisphere Figure 7) point to static B0 inhomogeneities. Apart from that, it should also be noted that the first‐order spherical harmonics field approximation in prospective shimming could even degrade image quality in certain brain regions if large motion occurs. Accordingly, prospective shimming may have also contributed to the shading differences visible in images of Subject 6.

In cases of minimal motion, blurring and shading artifacts were reduced in the frontal lobe, which can be attributed to the field drift correction. The precision of field estimates was increased by the moving average filter at the expense of delayed updates that decrease the effectiveness for removing respiratory oscillations, for example. Apart from other filters that could be tested to avoid or mitigate delayed updates, the $f_{0}$ precision could be increased by longer navigators, if imaging TE and TR allow for it. A delay correction of $f_{0}$ would also be possible retrospectively by demodulating the k‐space data with the difference of actual $f_{0}$ and the delayed $f_{0}$ corrected for at run‐time.

Considering the mapping of brain vasculature using susceptibility‐weighted imaging, all minIPs and venograms benefited from prospective corrections. Notably, the uncorrected SWI minIPs and QSM venograms provided more details than the uncorrected magnitude minIPs (cf. Figure 5). This may be related to the high‐pass filtered phase images involved in SWI and QSM. Improvements by servo navigation were nevertheless detectable. The vein‐tissue contrast could be further increased by shorter echo times at 7 T, as indicated in Figure 8. This is in agreement with the literature in which it was found to peak at 15 ms at 7 T [58]. At 11.7 T, even shorter echo times could be beneficial to enhance this contrast because of stronger $T_{2}^{*}$ decay. Preliminary results suggest that $T_{2}^{*}$ is approximately reduced by ∼30% at 11.7 T compared to 7 T (Stirnberg, Proc. ISMRM 2026).

To neglect $T_{2}^{*}$ blurring along the PE direction, the echo train length of the segmented 3D‐EPI readout should not exceed $T_{2}^{*}$ [59]. Although the echo train lengths of both protocols are quite different, they are approximately at this limit of their respective target field strengths (at 7 T: EPI factor 14 × ESP 2.4 ms; at 11.7 T: EPI factor 11 × ESP 2.32 ms). The strongly reduced $T_{2}^{*}$ at 11.7 T is demonstrated by the vastly different $T_{2}^{*}$ contrast observed in Subject 4 (7 T) and 7 (11.7 T), despite being scanned with almost identical Protocol II. Although all zoomed views of Subject 7 in Figure 8 demonstrate very high resolution, the actual voxel size along the anteroposterior PE direction was likely slightly larger than 0.3 mm. In future 11.7 T experiments, shorter echo trains or multi‐echo protocols should thus be explored by increasing the segmentation factor, which also enables $R_{2}^{*}$ mapping. Furthermore, multi‐echo acquisitions may be beneficial to increase the robustness against (dynamic) B0 inhomogeneities.

### 4.3 Impact

Servo navigation avoids many drawbacks of previous motion correction methods employed in mesoscopic imaging (e.g., marker‐based approaches or image‐space navigators [60]), allowing for high patient comfort, minimal sequence integration and high spatiotemporal precision. This may improve image quality and quantitative measures across various applications (e.g., anatomical imaging, SWI, QSM, fMRI).

Mesoscopic MRI is an emerging area of interest in neuroscience, with its potential and implications discussed in detail by Gulban et al. [61]. Recent advances, such as segmented 3D‐EPI acquisitions, have only recently made whole‐brain imaging at 0.35–0.4 mm isotropic resolution feasible within practical scan times. However, such studies have typically required well‐trained subjects, as even minor motion can compromise image quality at this resolution. The present work demonstrates that mesoscopic imaging can now be performed more robustly in untrained subjects, and potentially in patients, without added discomfort.

### 4.4 11.7 T Demonstration

Results of this work suggest that servo navigation can be employed at 11.7 T to increase the robustness against motion and field perturbations in acquisitions of 300 μm isotropic images. At unprecedented SNR levels [39], pursuing even higher isotropic resolutions of up to 100–200 μm appears feasible. Although the raw navigator precision is reduced in 3D‐EPI compared to less gradient‐demanding imaging sequences, the post‐filtering precision achieved here remains adequate for these resolutions. However, at this scale, non‐rigid pulsatile motion of the brain stem and mid‐brain, which occur on the same order of magnitude [62], may become a limiting factor. Mapping cortical regions and their vasculature may particularly benefit from whole‐brain coverage at high isotropic resolution. For example, detailed characterization of angioarchitecture may help to understand spatial variations in the BOLD signal [63].

## 5 Conclusion

In this study, servo navigation was explored to prospectively correct for motion and dynamic field changes in mesoscopic $T_{2}^{*}$‐weighted whole‐brain imaging at UHF. Servo navigation has consistently reduced motion‐ and field‐related artifacts at 7 and 11.7 T. In the presence of involuntary large motion, artifacts could be substantially mitigated. For microscopic motion, reduced blurring and shading were observed in fine vascular structures of the human brain, indicating an effective field drift correction. The integration of servo navigation into segmented 3D‐EPI suggests that rapid, motion‐ and field‐robust mesoscopic whole‐brain imaging at UHF may become feasible in untrained but cooperative subjects.

## Funding

This work was supported by the Horizon 2020 Framework Programme, Grant/Award Number: 885876; Agence Nationale de la Recherche (ANR) (Grant Number: ANR‐21‐ESRE‐0006).

## Conflicts of Interest

Klaas P. Pruessmann holds a research agreement with Philips Healthcare and is a shareholder of GyroTools LLC. Shajan Gunamony is a shareholder of MR CoilTech Limited, Glasgow, UK. The other authors declare no conflicts of interest.

## Supporting information


**Figure S1:** Retrospective analysis of motion and field estimate precision in vivo without processing (raw), after parameter bias correction, and after moving average filtering. The top panels show specific data for one example subject (Subject 6 that moved rapidly by ∼0.5° at 11.7 T); the bottom panels display the temporal standard deviations (∼1/precision) for all subjects. To minimize the influence of drifts and slow motion, a high‐pass filter was applied before computing the standard deviations. However, to still capture variations that vary along with the partition encoder (e.g., on a time scale of Segmentation factor *S* = 30 times TR = 43 ms ∼ 1.3 s for the 0.3 mm iso. protocol), the threshold of the high‐pass filter was set to 1/(S × TR) (e.g., 0.77 Hz), which can only exclude slow motions. Reduced precision can be observed in scans with pronounced motion (RMS deviation), either indicating more residual motion after high‐pass filtering, that is, more bias of the precision estimation, or reduced effectiveness of the proposed parameter bias correction. The estimation of the precision after filtering is restricted to frequencies between the high‐pass filter threshold as a lower bound (1/(S × TR)) and the run‐time moving average filter frequency as an upper bound (i.e., 1/(10 × TR) for motion, 1/(14 × TR) for field parameters).
**Figure S2:** Validation of servo navigation in phantom scans at 11.7 T with a 3D‐EPI time series protocol (TR = 58 ms, 36 shots/vol., TAvol = 2.12 s). (A): FoV updates and retrospectively registered motion parameters of an experiment in which the head phantom was moved abruptly by a stick from outside the scanner bore. Small amplitudes of residual motion (∼0.03°, 0.04 mm) demonstrate an effective motion correction despite relatively large actual motion (∼1.9°, 2.3 mm). (B): Registration parameters of the 3D‐EPI time series without and with prospective motion and field corrections as well as predicted and applied field updates of the “bottle” experiment (an air‐filled bottle was moved towards the head phantom). The apparent shift in y‐translation due to the introduced field change (and low PE bandwidth in y‐direction) is largely mitigated by prospective field corrections.
**Figure S3:** Axial, coronal and sagittal slices of 0.3 mm iso. images with and without servo navigation of volunteers 1–4 scanned at 7 T. Subjects 1–3 were scanned with Protocol I and Subject 4 with Protocol II that was designed for 11.7 T but applied with only minor changes at 7 T. Subject 2 exhibited the largest motion, leading to blurring artifacts without servo navigation.
**Figure S4:** SWI minIPs and QSM venograms of Subject 3. Reduced blurring artifacts of white matter medullary veins can be observed in the image of the corrected scan despite little motion in both scans.
**Figure S5:** Axial, coronal and sagittal slices of 0.3 mm iso. images with and without servo navigation of volunteers 5–7 scanned at 11.7 T. Strong blurring artifacts can be observed for Subject 6 who exhibited a large motion throughout and in particular during k‐space center acquisition in both scans. Although artifacts are reduced substantially in the corrected scan, residual blurring and shading remain.
**Figure S6:** Evaluation of the proposed bias correction in case of (rapid) large motion that is included in the sliding window (4 partitions = 192 shots) of the bias calculation. Data were simulated in python by generating a repeated bias pattern (segmentation factor 48) and adding Gaussian measurement noise. Different magnitudes of motion (0.5, 1, 2 mm) over fixed time intervals (2.6 s (48 shots) and 20.8 s (384 shots)) were evaluated. Rapid large pose changes cause temporary oscillations (e.g., ∼0.25 mm in the extreme case of a 2 mm motion per 48 shots). Slower motion causes smaller temporary oscillations, but if the sliding window is exclusively computed on large motion data (lower right plot), the motion slope is estimated as part of the bias and removed, which reduces the effectiveness of the method (resulting steps visible during the motion event). The temporary oscillations persist up to 192 shots (the sliding window size) after the large motion event has ended.

## Data Availability

Research data are not shared.
