# Supplementary material for: Motion‐ and Field‐Robust Mesoscopic Whole‐Brain T2*‐Weighted Imaging at 7 and 11.7 T Using Servo Navigation
Source: Magn Reson Med. 2026 Jan 12;95(5):2658–70. doi: 10.1002/mrm.70251 (PMC12962216; doi:10.1002/mrm.70251)

*Supporting Figure S1: Retrospective analysis of motion and field estimate precision in-vivo without processing (raw), after parameter bias correction, and after moving average filtering. The top panels show specific data for one example subject (Subject 6 that moved rapidly by ~ 0.5° at 11.7T); the bottom panels display the temporal standard deviations (~1/precision) for all subjects. To minimize the influence of drifts and slow motion, a high-pass filter was applied before computing the standard deviations. However, to still capture variations that vary along with the partition encoder (e.g. on a time scale of Segmentation factor S = 30 times TR = 43 ms ~ 1.3 s for the 0.3 mm iso. protocol), the threshold of the high-pass filter was set to 1/(S*TR) (e.g. 0.77Hz), which can only exclude slow motions. Reduced precision can be observed in scans with pronounced motion (RMS deviation), either indicating more residual motion after high-pass filtering, i.e. more bias of the precision estimation, or reduced effectiveness of the proposed parameter bias correction. The estimation of the precision after filtering is restricted to frequencies between the high-pass filter threshold as a lower bound (1/(S*TR)) and the run-time moving average filter frequency as an upper bound (i.e. 1/(10*TR) for motion, 1/(14*TR) for field parameters).*


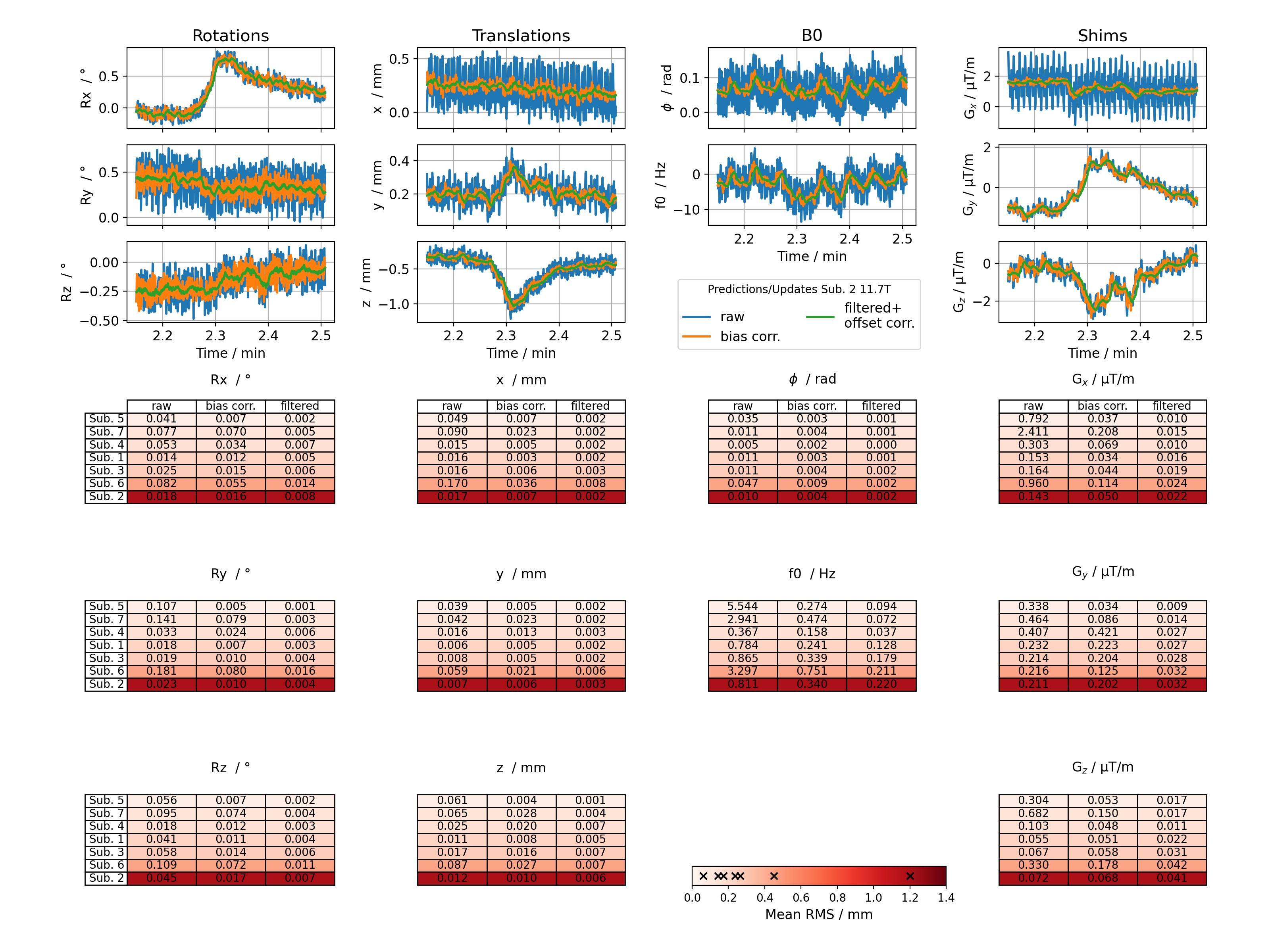


*Supporting Figure S2: Validation of servo navigation in phantom scans at 11.7T with a 3D-EPI time series protocol (TR=58ms, 36 shots / vol., TA_vol_ = 2.12 s). (A): FoV updates and retrospectively registered motion parameters of an experiment in which the head phantom was moved abruptly by a stick from outside the scanner bore. Small amplitudes of residual motion (~0.03°, 0.04 mm) demonstrate an effective motion correction despite relatively large actual motion (~1.9°, 2.3 mm). (B): Registration parameters of the 3D-EPI time series without and with prospective motion and field corrections as well as predicted and applied field updates of the “bottle” experiment (an air-filled bottle was moved towards the head phantom). The apparent shift in y-translation due to the introduced field change (and low PE bandwidth in y-direction) is largely mitigated by prospective field corrections.*


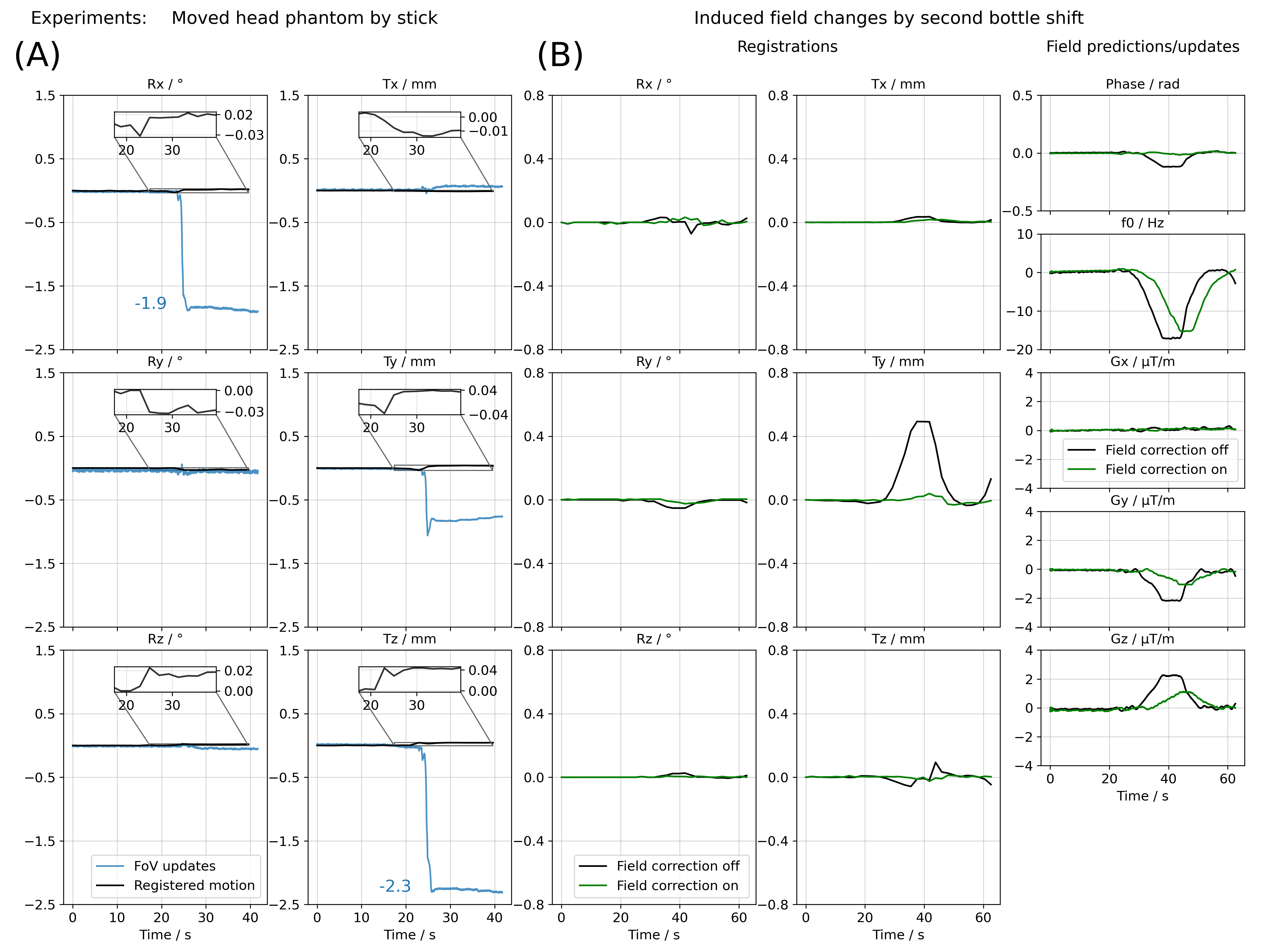


*Supporting Figure S3: Axial, coronal and sagittal slices of 0.3 mm iso. images with and without servo navigation of volunteers 1-4 scanned at 7T. Subjects 1-3 were scanned with Protocol I and Subject 4 with Protocol II that was designed for 11.7T but applied with only minor changes at 7T. Subject 2 exhibited the largest motion, leading to blurring artifacts without servo navigation.*
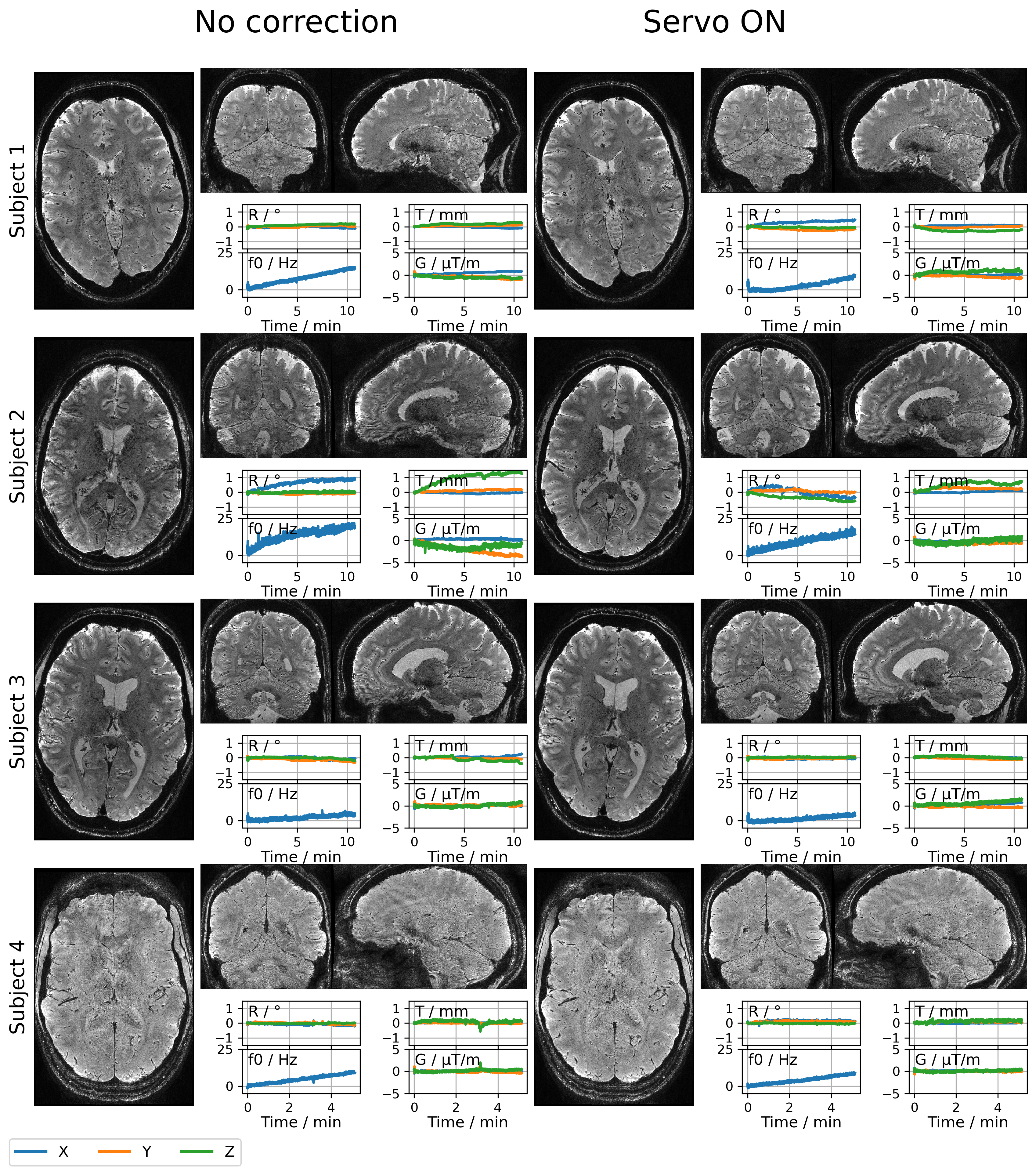


*Supporting Figure S4:* SWI minIPs and QSM venograms of Subject 3. Reduced blurring artifacts of white matter medullary veins can be observed in the image of the corrected scan despite little motion in both scans.


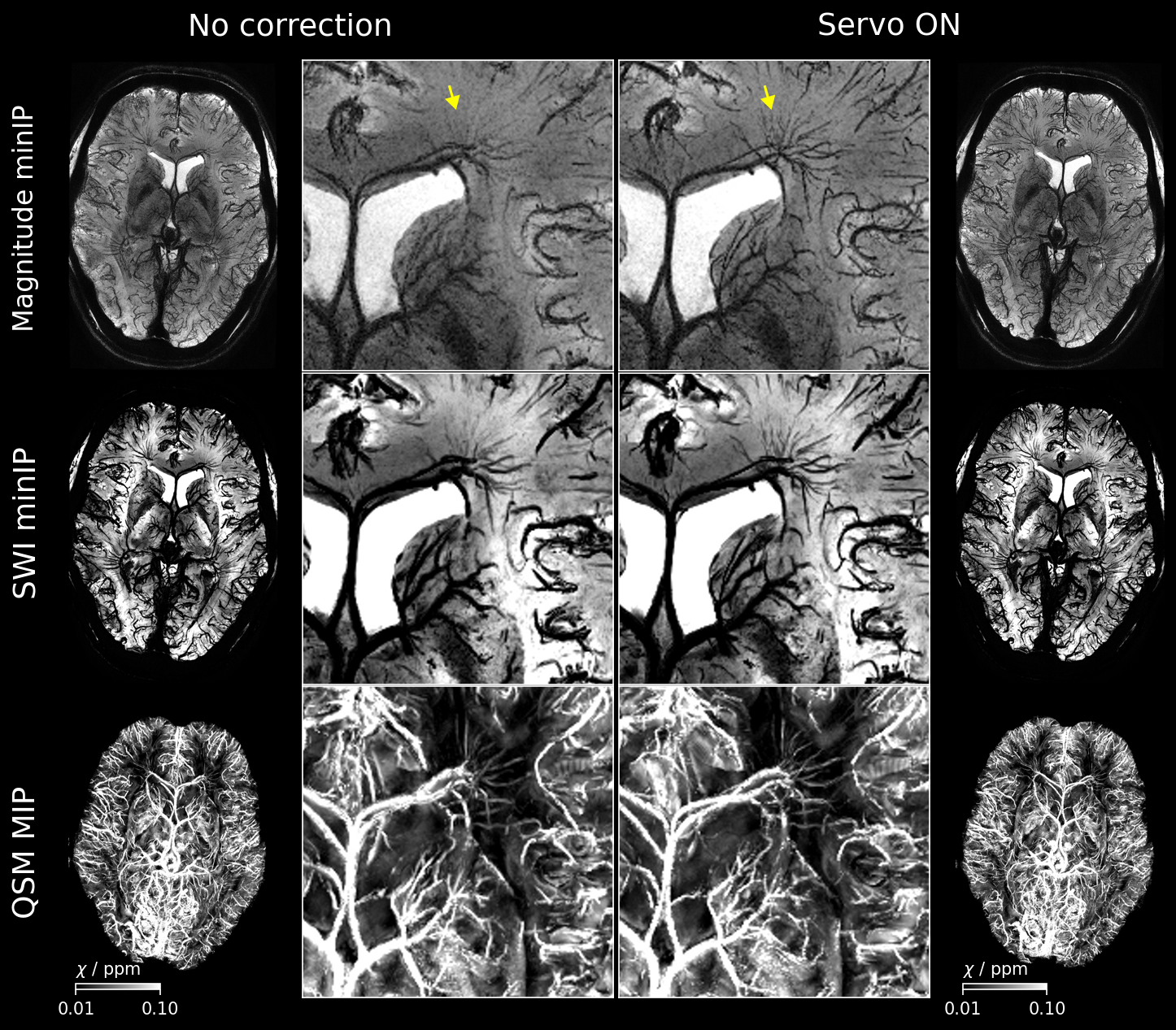


*Supporting Figure S5: Axial, coronal and sagittal slices of 0.3 mm iso. images with and without servo navigation of volunteers 5-7 scanned at 11.7T. Strong blurring artifacts can be observed for Subject 6 who exhibited a large motion throughout and in particular during k-space center acquisition in both scans. Although artifacts are reduced substantially in the corrected scan, residual blurring and shading remain.*


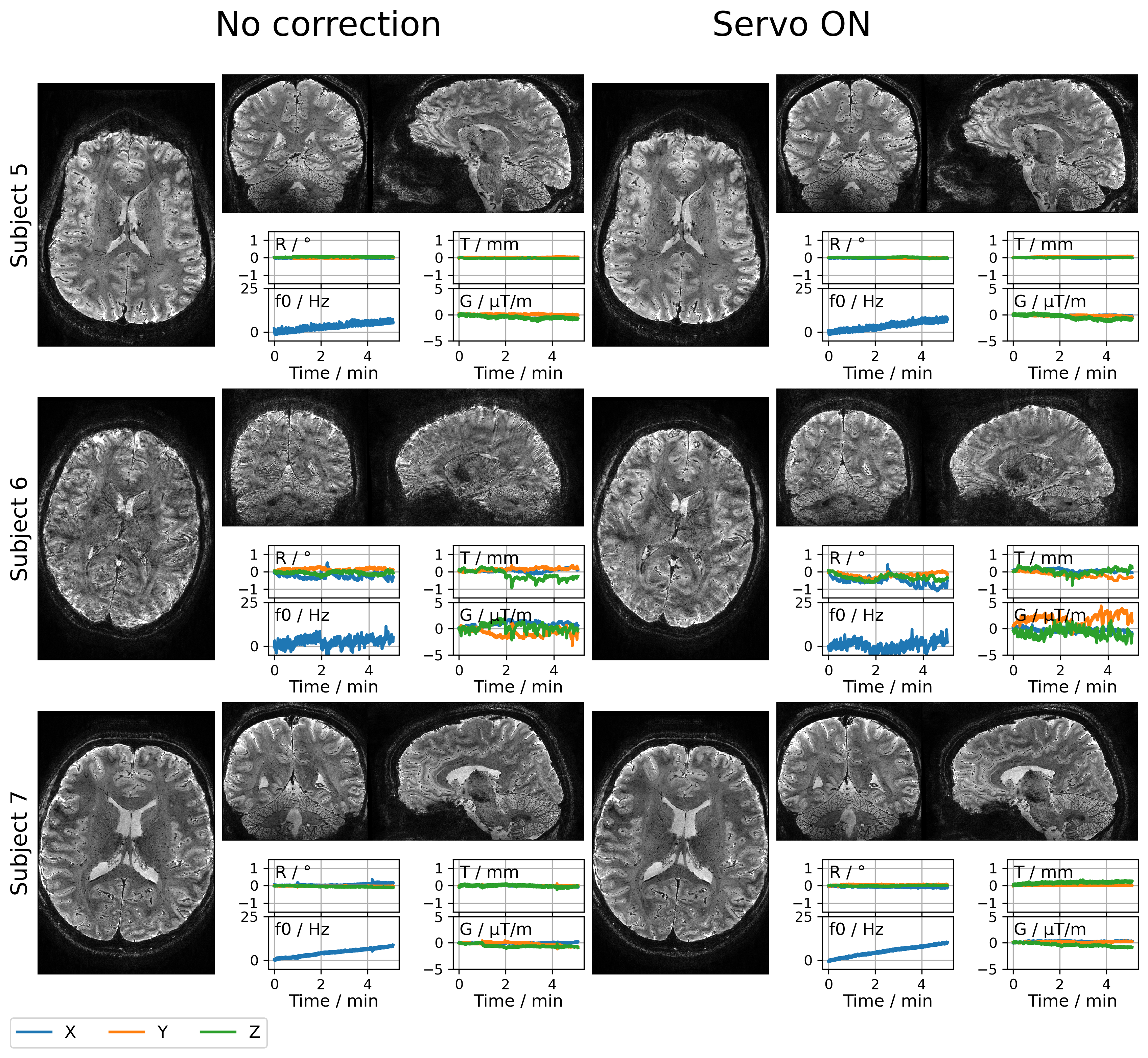


*Supporting Figure S6: Evaluation of the proposed bias correction in case of (rapid) large motion that is included in the sliding window (4 partitions = 192 shots) of the bias calculation. Data were simulated in python by generating a repeated bias pattern (segmentation factor 48) and adding Gaussian measurement noise. Different magnitudes of motion (0.5, 1, 2 mm) over fixed time intervals (2.6 seconds (48 shots) and 20.8 seconds (384 shots)) were evaluated. Rapid large pose changes cause temporary oscillations (e.g. ~ 0.25 mm in the extreme case of a 2 mm motion per 48 shots). Slower motion causes smaller temporary oscillations, but if the sliding window is exclusively computed on large motion data (lower right plot), the motion slope is estimated as part of the bias and removed, which reduces the effectiveness of the method (resulting steps visible during the motion event). The temporary oscillations persist up to 192 shots (the sliding window size) after the large motion event has ended.*


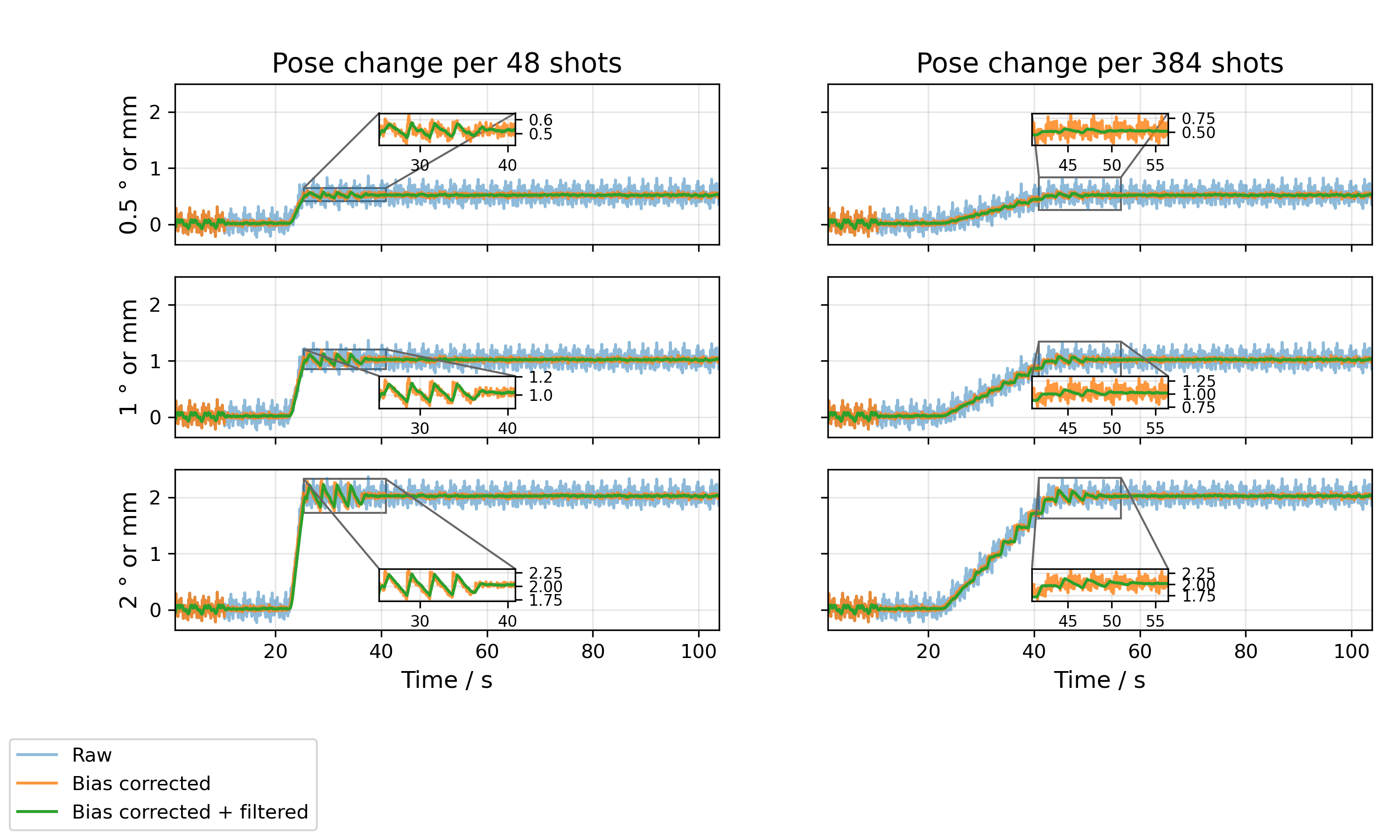

Supplement: Supplementary file 1 — Figure S1: Retrospective analysis of motion and field estimate precision in vivo without processing (raw), after parameter bias correction, and after moving average filtering. The top panels show specific data for one example subject (Subject 6 that moved rapidly by ∼0.5° at 11.7 T); the bottom panels display the temporal standard deviations (∼1/precision) for all subjects. To minimize the influence of drifts and slow motion, a high‐pass filter was applied before computing the standard deviations. However, to still capture variations that vary along with the partition encoder (e.g., on a time scale of Segmentation factor S = 30 times TR = 43 ms ∼ 1.3 s for the 0.3 mm iso. protocol), the threshold of the high‐pass filter was set to 1/(S × TR) (e.g., 0.77 Hz), which can only exclude slow motions. Reduced precision can be observed in scans with pronounced motion (RMS deviation), either indicating more residual motion after high‐pass filtering, that is, more bias of the precision estimation, or reduced effectiveness of the proposed parameter bias correction. The estimation of the precision after filtering is restricted to frequencies between the high‐pass filter threshold as a lower bound (1/(S × TR)) and the run‐time moving average filter frequency as an upper bound (i.e., 1/(10 × TR) for motion, 1/(14 × TR) for field parameters). Figure S2: Validation of servo navigation in phantom scans at 11.7 T with a 3D‐EPI time series protocol (TR = 58 ms, 36 shots/vol., TAvol = 2.12 s). (A): FoV updates and retrospectively registered motion parameters of an experiment in which the head phantom was moved abruptly by a stick from outside the scanner bore. Small amplitudes of residual motion (∼0.03°, 0.04 mm) demonstrate an effective motion correction despite relatively large actual motion (∼1.9°, 2.3 mm). (B): Registration parameters of the 3D‐EPI time series without and with prospective motion and field corrections as well as predicted and applied field updates of the “bottle” ex [file MRM-95-2658-s001.docx]
